# Virtual reality for chronic primary low back pain in adults: systematic review protocol

**DOI:** 10.1186/s13643-026-03307-6

**Published:** 2026-08-28

**Authors:** Axel Schäfer, Ulrike Kaiser, Alexander Elser, Christian Kopkow, Bernhard Elsner

**Affiliations:** 1 Faculty of Social Work and Health, University of Applied Sciences and Arts Hildesheim/Holzminden/Göttingen, Goschentor 1, Hildesheim, 31134 Germany; 2 Clinic for Anaesthesiology and Intensive Care Medicine, University Hospital Schleswig Holstein, Lübeck, Germany; 3 Department of Physiotherapy, Brandenburg University of Technology Cottbus-Senftenberg, Senftenberg, Germany; 4 Institute for Health Sciences, University of Luebeck, Lübeck, Germany; 5 Fraunhofer Research Institution for Individualized Medical Technology and Engineering IMTE, Luebeck, Germany

**Keywords:** Chronic primary low back pain, Virtual reality, Pain, Physical functioning, Systematic review, Meta-analyses

## Abstract

**Background:**

Chronic primary low back pain (LBP) is a costly and disabling condition, with complex interacting factors contributing to pain experience and disability. Although multimodal therapy, including physiotherapy, behavioural therapy/psychotherapy and education is recommended, implementation is limited due to shortcomings in healthcare delivery. Digital solutions such as virtual reality (VR) could offer possibilities to address these shortcomings and become increasingly common. However, the evidence base for VR in chronic LBP is conflicting. Nine existing meta-analyses show substantial variance in effect sizes, ranging from SMD 0.07 to 8.15, due to heterogeneity in population, interventions and outcome measurements, with most often limited quality of evidence. Therefore, a high-quality systematic review focussing on chronic primary LBP, incorporating mechanisms of action and state-of-the-art meta-analysis is warranted.

**Methods:**

We will conduct a systematic review, employing a comprehensive and sensitive search strategy in all relevant databases. We will include randomised controlled studies that investigate the effects of virtual reality versus other interventions (active and non-active) or no intervention on physical functioning, pain intensity and health related quality of life in patients with chronic primary low back pain. We will assess risk of bias with the Cochrane risk of bias tool 2 (RoB2) and certainty of the evidence with GRADE criteria. We will use a random-effects model for all statistical analyses.

**Discussion:**

This systematic review will provide trustworthy evidence about the effects of VR in chronic primary low back pain by employing rigorous methods of search string development, comprehensive database search, quality evaluation and control, data extraction, documentation and analysis incorporating theory of change. Anticipated intervention heterogeneity will be addressed by pre-specified subgroup analyses and appropriate methods of meta-analysis.

**Systematic review registration:**

PROSPERO 2025 CRD420251250648

## Introduction

### Rationale

Low back pain, which is characterised by ‘discomfort and pain situated below the ribcage and above the inferior gluteal folds, with or without leg pain’ [1], is highly prevalent, with serious consequences for those affected and resulting in significant direct and indirect costs. Chronic primary low back pain (CPLBP) accounts for as many as 90% of low back pain cases [2]. It is defined as pain in the lower back that persists or recurs for more than 3 months, without a consistent association with any underlying disease, structural damage, or deformity [2] and co-occurring substantial emotional distress—such as anxiety, anger, frustration, or depression—or functional impairment, which affects daily activities and participation in social roles [1]. Chronic primary LBP per definition is not attributable to one single pathoanatomical cause. Instead, a complex interaction of multiple bio-psycho-social factors determine individual pain experience and disability [3].

In 2020, the globally age-standardised prevalence rate^1^ of low back pain^2^ was 7.46% corresponding to estimated 619 million cases of low back pain with a 95% uncertainty interval ranging from 554 to 694 million [4]. Central Europe had the highest age-standardised prevalence rate of low back pain at 12.80% followed by Eastern Europe with a rate of 11.20%, and Australasia with 11.10. East Asia had the lowest age-standardised prevalence rate for low back pain with 5.43% [4]. In regards to sex, women are affected almost twice as often as men with a prevalence rate of 9.33% compared to males with 5.52% [4].

Compared to the combined data of all ages and sexes in 1990, a 60.4% increase of prevalent cases (ranging from 57.1 to 64.2%) was observed by 2020 and projection estimates indicate that the global prevalence rate will only decrease slightly by 0.22% until 2050 [4]. As the number of prevalent cases and years lived with disability (YLDs) due to low back pain rise with age, reaching their highest point in the 85–89-year age group, this trend poses significant challenges for countries with ageing populations [4], such as western Europe, northern America and Japan [5]. From the perspective of those affected, low back pain was the leading cause for years lived with disability (YLDs) worldwide, with only a slight reduction of 2.4% (ranging from 1.8 to 2.9%) in the age-standardised YLD rate since 2010 [6].

In spite of technological and scientific advances in medicine and rehabilitation and the exponentially increasing research output in relation to low back pain, little progress has been made to curb prevalence rates and to provide effective therapies on a sufficient scale. The WHO recommends education, exercise, behavioural therapy, medication (NSARs) and multicomponent biopsychosocial care for the treatment of persons with chronic primary low back pain [2]. However, even in high income countries, access to recommended, high-quality therapies is constrained due to limited availability and reimbursement [7]. eHealth applications, such as VR, may offer a broader availability of these recommended interventions, including exercise therapy, education and behaviour-based interventions. VR is defined as a three-dimensional computer-generated simulated environment that replicates real-world or imaginary environments and interactions to support work, education, recreation, and healthcare [8].

Different mechanisms of effect have been described for the treatment of chronic pain using VR. One mechanism is distraction, where attention of patients is redirected from pain by engaging them in multisensory experiences [9]. Another mechanism is graded exposure, where VR helps patients to confront pain-related fears, such as kinesiophobia, in controlled environments [10, 11]. Embodiment as one other mechanism relates to the experience of altered body representations, which can help reframe pain perception and improve motor function by increasing the sense of body ownership and agency [10, 11]. In this way, VR can facilitate neuromodulation by altering cortical activity and promoting neuroplasticity, which may reshape pain perception and improve body awareness [12, 13]. VR-delivered relaxation exercises may induce physiological mechanisms such as muscle relaxation, reduction of heart rate and respiratory activity, as well as psychological mechanisms by inducing calm, serenity and well-being. Given the multiple mechanisms of action of VR delivered interventions, a theory of change that explains the relationship between pathophysiological mechanisms, intervention components and outcomes will be used to guide this systematic review.

Bibliometric analysis shows that VR is a trending research topic in recent years for the management of pain [14]. It is not surprising, therefore, that there are systematic reviews that evaluate the effects of VR in the treatment of low back pain. We found 9 systematic reviews with meta-analysis [15–23] evaluating effects of VR in patients with chronic low back pain, showing significant and non-significant effect sizes (standardised mean difference, SMD) for pain reduction ranging from 0.07 to 8.15. For example, Li et al. [21] concluded on the basis of 20 identified trials, that VR-based training can decrease pain (SMD = 0.22) immediate-term (defined as the period after intervention up to 3 months), but not in the short term (3–6 months) (SMD = 0.07). In another systematic review including 11 studies, a large significant effect on pain reduction (SMD = 1.92) was found for VR versus no VR immediately after the intervention and at 6 months post-intervention (SMD = 6.34) [16]. Taken together, the results from identified systematic reviews with meta-analysis are inconclusive, with a large variety of in- and exclusion criteria, comparisons and mostly insufficient systematic rigour. The key deficiencies in methodology of these previously published systematic reviews include:

- Inclusion criteria are too broad (e.g. chronic musculoskeletal pain, chronic pain, pain) leading to greater heterogeneity and unspecific interpretation of findings [15, 17–19, 22, 24]
- Risk of bias assessment of included studies flawed (e.g. using unsuitable risk of bias assessment tools) [17]
- No or incomplete evaluation of quality of evidence across outcomes following the GRADE framework [16, 17, 20, 24]
- Number of databases searched is limited [16]
- Restriction to English language [17, 20, 21, 24]

As VR is a rapidly developing technology, we also expect that new studies have been published in the meantime that will be included in our systematic review.

To resolve the ambiguous and conflicting evidence base, we will conduct a high-quality systematic review with a specific research question, incorporating theory of change, a sensitive comprehensive search strategy, rigorous assessment of evidence quality and meta-analysis.

## Objectives

The objective of this review is to evaluate the effects of virtual reality (VR) on physical functioning, pain intensity and health-related quality of life, as well as the harms associated with it, in adults with chronic primary low back pain, by comparing VR with other interventions (active and inactive).

## Methods

### Eligibility criteria

We will include all randomised studies examining the effects of virtual reality on improving chronic primary low back pain in adults.

#### Types of studies

We will include genuine randomised controlled trials (RCTs) or randomised cross-over trials from 1980 onwards. From the latter, we will only analyse first-period data until the wash-out phase. We will also include cluster-randomised trials.

#### Types of participants (Table 1)

**Table 1:** Participants

Characteristic	Inclusion criteria of review	Anticipated representation in included studies (people we expect to see)
Age	18–80 years	18–80 years
Sex	all	Male, female
Socio-economic status	all	all
Level of education	all	all
Ethnicity	all	all
Setting	all	Primary care, rehabilitation
Location (country/countries of data collection)	all	High-, mid-, and low-income countries

We will include all studies with adults (≥ 18 years) reporting primary chronic LBP from high, mid and low income countries. Primary chronic pain (ICD-11 MG 30.02), formerly known as non-specific LBP, is defined as pain in the lower back that persists or recurs for more than 3 months, without a consistent association with any underlying disease, structural damage, or deformity [2]. Participants will be eligible from any setting. We will not exclude studies on the basis of socioeconomic status, level of education or ethnicity.

#### Types of interventions

We will include all studies which applied VR devices and systems of the following three categories based on the extent of immersion: low-immersive, moderate-immersive, and high-immersive according to the classification of Bordeleau et al. [23]:

- Low-immersive systems use desktop monitors or handheld displays to provide a limited field of view of the virtual environment. They are not associated with tracking systems and the visual experience does not need to match proprioceptive feedback.
- Moderate-immersive systems may involve a large and concave screen with a projection system that provides the user with a wide field of view, similar to a large screen cinema experience. They may be associated with a tracking system.
- High-immersive systems consist of a head-mounted display or surround screen that completely fills the user’s field of view. They incorporate a body motion capture system to closely match the visual experience with proprioceptive feedback for optimal sensorimotor integration.

All categories are included, either as stand-alone therapy or in combination with other therapies. For comparisons between different types of virtual reality we will include co-interventions, including exercise interventions, provided that they are evenly distributed between groups.

#### Types of comparators

Active interventions and inactive interventions (Table 2). Active Interventions are treatments or therapies that are intended to have a specific therapeutic effect on the condition, such as physiotherapy, exercise programmes, medication, cognitive-behavioural therapy, manual therapy etc. Inactive Interventions are treatments or procedures that are not expected to have a specific therapeutic effect, for example placebo treatments (e.g. attention placebo, sham procedure, inactive medication), educational leaflets without specific therapeutic content, general health advice not targeting back pain, etc. [25]. Inactive interventions may also include no intervention, meaning that participants in this group do not receive any form of treatment or placebo during the study period, for example waiting list control (participants do not receive treatment until after the study period) or usual care, if it implies no specific intervention for the condition [25].

**Table 2:** Classification of control interventions with examples according to the Cochrane Handbook Section 3.2.2 [25]

Active control intervention	Inactive control intervention
Exercise	Attention placebo
Medication	Sham procedure
Cognitive behavioral therapy	Inactive medication
Manual therapy	Unspecific educational leaflets
Usual care with specific intervention components for the condition	Unspecific general health advice
	Usual care without specific intervention components for the condition
	Waiting list control

Active interventions: specific mechanism of action

Inactive interventions: non-specific or no mechanism of action

#### Outcomes

Physical functioning, pain intensity and health related quality of life.

### Information sources

We will search the following electronic bibliographic databases. The search will be restricted to studies published from 1980 because previously VR was not commercially available [26].

- PubMed (from 1980)
- Cochrane Central Register of Controlled Trials (CENTRAL; latest issue), in the Cochrane Library
- MEDLINE in Ovid (from 1980)
- Embase in Ovid (from 1980)
- PsycInfo (from 1980)
- PsycArticle (from 1980)
- Cumulative Index to Nursing and Allied Health Literature (CINAHL) in EBSCO (from 1982)
- Allied and Complementary Medicine Database (AMED) in Ovid (from 1985)
- Web of Science (Science Citation Index Expanded, Social Sciences Citation Index, Arts and Humanities Citation Index) (from 1980)
- Scopus (from 1980)
- Physiotherapy Evidence Database (PEDro)
- COMPENDEX (from 1980)
- Inspec (from 1980)

We will search the World Health Organization International Clinical Trials Registry Platform (WHO ICTRP), at https://trialsearch.who.int as well as US National Library of Medicine (https://clinicaltrials.gov) for ongoing trials.

Additionally, we will review the reference lists of included studies and related systematic reviews.

#### Searching other resources

We will contact equipment manufacturers and experts in the field in order to identify further published and unpublished studies. We will use forward citation search in the Web of Science. We will exclude reports from conference proceedings.

### Search strategy

We will develop the search strategies with the help of a medical computer scientist (BE) and adapt the MEDLINE search strategy (Table 3) for use with the other databases. We will use the Cochrane highly sensitive RCT-filter [27].

**Table 3:** Search strategy Medline via Ovid

1. *Back Pain/or *Sciatica/or (*Chronic Pain/or *Low Back Pain/) or ((chronic adj1 pain) OR (low* adj1 back adj1 (ache or pain)) or lumbago or sciati*).tw
2. (lumb* or radic* or (nerve adj1 root*) or sacroiliac* or spin* or dis?? or vertebr*).tw
3. 1 OR 2
4. user-computer interface/
5. computers/or exp microcomputers/or computer systems/or software/
6. computer simulation/or computer-assisted instruction/or therapy, computer-assisted/
7. computer graphics/or video games/or *touch/
8. (virtual reality$ or virtual-reality$ or VR).tw
9. (virtual adj3 (environment$ or object$ or world$ or treatment$ or system$ or program$ or rehabilitation$ or therap$ or driving or drive$ or car or tunnel or vehicle)).tw
10. (computer adj3 (simulat$ or graphic$ or game$ or interact$)).tw
11. (computer adj1 assist$ adj1 (therap$ or treat$)).tw
12. (computer adj1 generat$ adj1 (environment$ or object$)).tw
13. (video game$ or video gaming or gaming console$ or interactive game or interactive gaming or Nintendo Wii or gaming program$).tw
14. (haptics or haptic device$).tw
15. (simulat$ adj3 (environment$ or object$ or event$1 or driving or drive$ or car or tunnel or vehicle)).tw
16. (user adj1 computer adj1 interface).tw
17. or/4–16
18. exp randomized controlled trial/
19. controlled clinical trial.pt
20. randomized.ab
21. placebo.ab
22. drug therapy.fs
23. randomly.ab
24. trial.ab
25. groups.ab
26. or/18–25
27. 3 and 17 and 26
28. exp animals/not humans.sh
29. 27 not 28

### Study records

#### Data management

We will manage the results of our search with Endnote [28].

#### Selection process

Two review authors (AE, BE) will independently read the titles and abstracts of identified references and will eliminate obviously irrelevant studies. We will obtain the full text for the remaining studies. Based on our inclusion criteria, the same two review authors will independently categorise these studies as relevant, irrelevant, or possibly relevant. We will exclude all trials ranked initially as irrelevant but include all other trials at this stage. We will resolve any disagreements through discussion between all review authors. If we require further information to reach consensus, we will contact trialists in an attempt to obtain the missing information. We will record the selection process in sufficient detail to complete a PRISMA flow diagram, and we will list in the excluded studies table all studies that will not match our inclusion criteria, with reasons. If one of the review authors was a co-author of an included trial, another review author will make eligibility decisions.

We will use the artificial intelligence (AI) supported software ASReview [29] in order to speed up the screening and data extraction process. This software is able to pre-sort the list of database entries found according to their highest expected probability of being relevant. The reliability of ASReview is supported by the validation study of van de Schoot et al. [29], which demonstrated through simulation studies that its active-learning approach can substantially reduce screening workload while maintaining high recall and high-quality identification of relevant studies. This pre-sorted database set will be screened by two reviewers (AE, BE).

We will not include or exclude publications of studies based on language. In the case of publications in languages other than English or German we will use our personal network to identify knowledgeable native speakers or use automated translation software such as DeepL [30] to assist translation.

Post-publication amendments published on included or eligible studies, such as expressions of concern, errata, corrigenda and retractions, will also be considered.

#### Data collection process

Two of the following review authors (AS, AE, UK, BE) will independently extract trial and outcome data from the selected trials. We will establish the characteristics of unpublished trials through correspondence with the trial co-ordinator or principal investigator. If any review author was involved in any of the selected studies, another review author not involved in the study will extract the study information. We will use checklists to independently record the variables.

The two review authors will check all the extracted data for agreement, with a third review author (CK) arbitrating any items for which consensus could not be reached. If necessary, we will contact trialists to request more information, clarification, or missing data.

### Data items

Data for the following variables will be extracted:

- Methods of generating the randomisation schedule.
- Method of concealment of allocation.
- Blinding of assessors.
- Use of an intention-to-treat analysis (all participants initially randomly assigned were included in analyses as allocated to groups).
- Adverse events and dropouts for all reasons.
- Important imbalance in prognostic factors.
- Participants (country, ethnicity, number of participants, age, sex, socio-economic status, form of pain (primary vs. secondary), time from disease onset to entry to the study, inclusion and exclusion criteria).
- Comparison (details of the intervention in treatment groups, details of the intervention in control groups classified as active and inactive, details of co-intervention(s) in both groups, duration of treatment, disease severity, age, sex, education level, intensity of treatment per day, dropouts, funding).
- Outcomes and time points of measures (number of participants in each group and outcome, regardless of compliance). Time points of measure will be categorised in short, medium, and long-term follow-up.
- Mechanisms targeted by the intervention in the VR environment according to Table 4. Table 4 depicts the relation between pathophysiological mechanisms (biophysical, psychological, social and lifestyle), therapeutically addressed pathomechanism, mechanism of the intervention and outcome. For example, one biophysical factor contributing to chronic primary LBP is altered central pain processing and modulation [3]. The therapeutically addressed pathomechanism is central sensitisation, the mechanism of the intervention is improvement of endogenous pain modulation and the outcome is reduction of pain intensity and sensitivity.

**Table 4:** Pathomechanisms, mechanisms of intervention, and outcome

Pathomechanism/contributing factor	Therapeutically addressed pathomechanism	Possible mechanism of intervention	Outcome Domain	Possible outcome measure
Biophysical factors				
Muscle structure	Muscle atrophy and increased fat content [31, 32]	Hypertrophy and reduction of fat content	Muscle diameter (increase) Fat content (decrease)	Diameter in cm, percentage of fat content
Posture and movement	Impaired movement coordination and proprioception of the body, esp. the back [33]	Improve coordination and proprioception	Economic and physiological posture and movement	Two point discrimination (cm), Joint position sense (cm), extent of mastering a movement task
Physical load	Increased structural load due to biomechanical changes [34]	Reduction of structural load	Force acting on joints, ligaments and muscles	Torque
Central pain processing and modulation	Central sensitisation [35] Nociplastic pain [36]	Improve endogenous pain modulation [37]	Pain reduction	Pain detection threshold Change in pain intensity (self report)
Brain structure	Structural neuroplasticity, structural gray and white matter changes [38]	Normalisation of structural neuroplasticity and grey/white matter composition	White and grey matter increase	Volume (mm^3^) Density cm^3^/g
Brain function	Functional connectivity [38]	Normalisation of functional connectivity	Decrease activity in pain related brain regions und increase activity in analgesic regions	blood flow and oxygenation in the brain (cerebral blood flow, blood oxygenation)
Genetics	not expected target of VR delivered interventions			
Psychological factors				
Depression [39]	Negative affect as a manifestation of mood state	Emotional regulation	Extent of depressiveness (reduction) Extent of emotion regulation (increase) Extent of emotion variability (increase)	Amount of symptoms of depression and anxiety related thoughts, feelings, moods, and behaviours Amount of different emotions in daily living for both, aversive and comfortable emotions
	Increase of help- and hopelessness [40]	Regaining mental control	Extent of self-regulation (increase) Extent of experienced action control (increase)	Amount of action orientation: The ability to remain capable of acting despite stress or failure Situation orientation: the tendency to focus on problems or negative experiences, which can impair the ability to act Emotional self-regulation: the ability to control emotions and remain calm in stressful situations
	Experience of stress/distress [41]	Physiological relaxation response Increase in parasympathetic nervous system activity and reduction in sympathetic nervous system activity	Extent of heart rate (reduction) Extent of muscular tension (reduction) Extent of respiratory rate (reduction)	Heart rate Muscle tension (back, low back) Respiratory rate
		Re-evaluation and reframing of threatening situations (physical movement in case of pain)	Extent of catastrophising (reduction)	Amount of symptoms of catastrophic thinking (magnification, rumination, helplessness)
States of anxiety	Catastrophising [42], fear-avoidance beliefs (see there)		Extent of movement-related anxiety (reduction)	Amount of thoughts and behaviours driven by fear of pain related cognitive patterns
	Negative evaluation of pain experience in relation to development over time, creation of helplessness [43]	Reconceptualisation of internal resources (physical, psychological) for coping with the experience of pain and impairment	Extent of helplessness (reduction) Extent of depression (reduction) Extent of anxiety (reduction)	Amount of symptoms of depression and anxiety related thoughts, feelings, moods, and behaviours
Catastrophising [42]	Negative assessment of the connection between pain and movement and the corresponding consequences [44]	Reconceptualisation of movement and pain	Extent of catastrophising (reduction)	Amount of symptoms of catastrophic thinking (magnification, rumination, helplessness)
Self-efficacy [45]	Lack of individual coping resources	Practicing individual coping resources (e.g. directing attention, relaxation)	Extent of self-efficacy experience (increase)	Amount of thoughts related to a person's belief in their ability to achieve desired outcomes or overcome challenges through their own actions
Fear-avoidance beliefs [46, 47]	Negative assessment of the connection between pain and movement and the corresponding consequences [44, 48, 49]	Reconceptualisation of movement and pain	Extent of catastrophising (reduction) Extent of movement-related anxiety (reduction)	Amount of thoughts and behaviours driven by fear of pain related cognitive patterns
Social factors				
Low income [50]	Not expected target of VR delivered interventions, but needs to be considered as a contributing factor for implementation and outcome			
Low level of education [46, 50, 51]				
satisfaction with work [46]				
High physical work load [46]				
Lifestyle factors				
Smoking [46]	Not expected target of VR delivered interventions, but needs to be considered as a contributing factor for implementation and outcome			
Body weight [46, 51]				
Low physical activity [52]

### Outcomes and prioritisation

Critical outcomes: for low back pain a Core Outcome Set is available [53]. It recommends for all clinical trials addressing low back pain the following outcome measures: physical functioning, pain intensity and health related quality of life, that will be considered as critical outcomes in this review.

Important outcomes: we will gather all biophysical and psychological outcomes in relation to pathophysiological mechanisms targeted by the VR intervention as reported by the authors (for expected outcome domains and examples of outcome measures see Table 4).

We will also consider deaths, adverse events, other forms of harm reporting and withdrawal from treatment.

### Risk of bias in individual studies

Two review authors (AS, BE) will independently use the Cochrane risk of bias tool 2 (RoB2) and its corresponding extensions for randomised cross-over and cluster randomised trials for assessing bias in all included studies. We are interested in quantifying the effect of assignment to the interventions at baseline, regardless of whether the interventions are received as intended (the ‘intention-to-treat effect’).

We will also assess risk of bias on outcome level across studies for our primary outcome measures at the end of intervention and at follow-up (> 3 months after the end of intervention). This is done independently by two review authors (AS, BE). If one of the review authors was a co-author of an included trial, another review author will make risk of bias assessments for that trial. If there is insufficient information to assess risk of bias, we will contact trialists in order to receive the missing information. Any disagreements between reviewers will be resolved by consulting a third reviewer (CK).

### Data

#### Synthesis

Two authors (BE and AS) will judge whether it is appropriate to perform a meta-analysis based on the amount of clinical diversity among subgroups of studies. In case of disagreement, a third review author (CK) will be consulted. We will use a random-effects model for all statistical analyses since we expect there will be clinical diversity. The Restricted Maximum Likelihood (REML) estimator will be used to estimate between-trial variance. The Hartung-Knapp-Sidik-Jonkman method will be used to calculate a confidence interval for the meta-analysis effect estimate when there are at least 3 studies, and the estimate of heterogeneity is greater than zero. In other scenarios (i.e. in pooled analyses of 2 studies, or where the estimate of heterogeneity is equal to zero) we will use the Wald-type method [54]. For dichotomous variables, we will calculate and report odds ratios (ORs) with 95% confidence intervals (CIs). For continuous data, we will calculate the treatment effect using standardised mean differences (SMDs) and 95% CIs when studies used different scales for assessment of the same outcome, and we will use mean differences (MDs) and 95% CIs when all studies used the same method of measuring an outcome. We will use RevMan 5.4 software for all statistical comparisons [55]. We will also judge whether effects reach the minimal important difference, as established for the respective outcome measures. If a minimal important difference threshold has not been established for a particular outcome measure, the authors will consider alternative approaches to interpret the clinical relevance of the findings. Depending on the outcome measure, this could include referencing MID values from similar or related outcome measures, or expert consensus to estimate an approximate MID, or use a rule of thumb relating to at least moderate effect sizes for clinical relevance. In the discussion section, limitations arising from the absence of an established MID will be acknowledged.

#### Unit of analysis issues

We anticipate that the majority of trials will have a parallel-group design. If studies have two or more active intervention groups eligible for inclusion within the same comparison (against a control, placebo, or no treatment group), we intend to 'share' control group data between the multiple pairwise comparisons to avoid double counting of participants within an analysis. If studies have used a randomised controlled crossover design, we will analyse data from the first phase only (up to the point of crossover). For cluster-randomised controlled trials, we will use the method of effective sample size incorporating the number and size of clusters and an estimate of the intracluster correlation coefficient (ICC) to calculate the design effect and effective sample size [56].

#### Dealing with missing data

In the case of missing outcome data, we will contact the trial coordinator or principal investigator. If study authors do not respond to requests for additional information we will analyse only the available data.

#### Synthesis methods

We will include studies of virtual reality interventions delivered in any form (e.g. immersive, semi-immersive, non-immersive) with the following comparisons.

1. Virtual reality vs. active interventions,
2. Virtual reality vs. inactive interventions

For comparisons between different types of virtual reality we will include co-interventions, including types of exercise interventions, provided that they are evenly distributed between groups.

#### Investigation of heterogeneity

We will use the *I*^2^ statistic as implemented in RevMan to assess heterogeneity. We will consider *I*^2^ > 50% as showing substantial heterogeneity. If *I*^2^ > 50%, we will explore individual trial characteristics to identify potential sources of heterogeneity. We will use a random-effects model, regardless of the level of heterogeneity. Additionally, we will consider possible clinical and methodological heterogeneity across studies when interpreting pooled estimates.

#### Subgroup analysis

If there is sufficient data (> 10 studies), we will perform a formal subgroup analysis for our primary outcomes following the guidance in the Cochrane Handbook for Systematic Reviews of Interventions [54], comparing subgroups with each other. We will assess subgroups using the formal test for subgroup differences implemented in RevMan.

We plan to carry out the following subgroup analyses for the primary outcomes pain intensity and physical functioning:

- Level of immersion (low-immersive, moderate-immersive, and high-immersive) [23]
- Age (median split of the mean age reported in included studies) [57]
- Sex (women vs. men)
- Level of pain intensity (< 4 vs. ≥ 4 on rating scale ranging from 0 to 10) [57]
- Socio-economic status: between high-middle and low income groups. The low income group has less than 67% of the median disposable income of a country’s total population, the middle income group between 67 and 200% and the high income group more than 200% of the median [58].
- Type of control group [57] (active vs. inactive interventions; other interventions including distraction vs. other intervention without distraction)
- Follow-up duration: short-, medium- and long-term follow-up
- Setting (e.g. home-based vs. clinic, supervised vs. unsupervised, primary care vs. tertiary care)
- Country setting (LMICs vs. HICs) to explore differences in effect between high- and low income countries as one aspect of health equity. Countries are classified by gross national income per capita [59]: Low income: $1135 or less; Lower-middle income: $1136 to $4495, Upper-middle income: $4496 to $13,935 and High income: More than $13,935.

We will gather information about the degree of immersion, the type of intervention provided by VR and the mechanisms targeted by the intervention in the VR environment according to Table 4.

#### Equity-related assessment

This review will address issues of health equity. VR may provide access to pain education, cognitive and behavioural therapy that would otherwise not be easily available, for example in remote rural areas or in socially disadvantaged communities. On the other hand, access to VR interventions may be more limited in LMICs compared to HICs. We will therefore incorporate subgroup analyses to explore differences in effect between high- and low-income countries, high-middle vs. low-income groups and between women and men.

#### Sensitivity analysis

We will carry out sensitivity analyses by including only studies with low risk of bias. A low risk of bias study is defined as one having low risk of bias for all domains of the RoB2 tool [60].

We anticipate that quantitative pooling of data is possible. However, clinical and methodological heterogeneity may require additional qualitative synthesis.

#### Meta-bias(es)

Two review authors (BE, AS) will inspect funnel plots to assess the risk of publication bias. Funnel plots are only used if there are 10 or more studies in an analysis [61].

#### Confidence in cumulative evidence

We will create a ‘Summary of findings’ table using the primary outcome measures ‘physical functioning’, ‘pain intensity’, and ‘health-related quality of life’ to assess the quality of evidence.

- At the end of the intervention phase, all VR types were used.
- At follow-up after study end (> 3 months).

Two review authors (BE, AS) will use the five GRADE criteria (study limitations, consistency of effect, imprecision, indirectness, and publication bias) to independently assess the certainty of the evidence [62]. Differences in opinions will be resolved by consulting a third reviewer (CK). We will use the methods and recommendations described in Chapter 14 of the Cochrane Handbook for Systematic Reviews of Interventions [63], employing GRADEpro GDT software [64]. We will justify all decisions to downgrade the certainty of the quality of evidence of studies using footnotes, and make comments to aid the reader's understanding of the review where necessary. If one of the review authors was a co-author of an included trial, another review author will make GRADE assessments for that trial. We will consult the recently published series of ‘Core GRADE’ papers by Guyatt et al. [65] as a resource for decision-making on downgrading evidence and potential terminology for plain language summaries in the summary of evidence tables.

## Discussion

This systematic review will provide trustworthy evidence about the effects of virtual reality in chronic primary low back pain. This will be achieved by following a rigorous methodology as recommended in the Cochrane Handbook [66]. In particular, this will include:

- Narrow inclusion and exclusion criteria to reduce heterogeneity
- Comprehensive search strategy in all relevant databases
- No language restrictions
- Evaluation of Risk of Bias of included studies using the RoB2 tool
- Evaluation of the quality of evidence across studies for each outcome using the GRADE approach
- Meta-Analyses including pre-planned subgroup analyses
- Employing theory of change to inform subgroup analyses and interpretation of findings (Table 4)

In spite of narrow inclusion and exclusion criteria, a considerable heterogeneity is anticipated, in particular regarding a broad range of different VR-based interventions, technology and settings. This will be addressed by employing random effect models in the meta-analysis and by subgroup analyses.

Also, the number of planned subgroup analyses is relatively large. Although we anticipate a sufficient number studies to be included in this systematic review, for some of the subgroup analyses statistical power might not be sufficient which may lead to limitations of the interpretation.

## Data Availability

No datasets were generated or analysed during the current study.

## Abbreviations

CI: Confidence interval
GRADE: Grading of Recommendations, Assessment, Development and Evaluation
HIC: High-income country
LBP: Low back pain
LMIC: Low- to middle-income country
NSAR: Non-steroidal anti-inflammatory drug
OR: Odds ratio
RoB2: Cochrane Risk of Bias Tool 2
SMD: Standard mean difference
VR: Virtual reality
WHO: World Health Organisation
YLD: Years lived with disability

## Footnotes

1 Prevalence rate: *P* = 1000 × npresent/n (Riffenburgh RH. Chapter 9—Epidemiology. In: Riffenburgh RH, editor. Statistics in Medicine (Second Edition). Burlington: Academic Press; 2006. p. 137–51)

2 ‘The case definition for low back pain was pain in the posterior aspect of the body from the lower margin of the 12th ribs to the lower gluteal folds, with or without pain referred into one or both lower limbs, and lasting for at least 1 day.’ GBD 2023.
